# How safe it is to treat pregnant FMF patients with Anakinra?

**DOI:** 10.1186/1546-0096-13-S1-P124

**Published:** 2015-09-28

**Authors:** H Ozdogan, S Ugurlu, B Ergezen

**Affiliations:** 1 Cerrahpasa Medical Faculty, University of Istanbul, Division of Rheumatology, Department of Internal Medicine, Istanbul, Turkey

## Background

It has been reported that anakinra, an anti-IL-1R antagonist, may be a safe alternative during pregnancy in patients with various autoinflammatory syndromes (1,2).

## Objectives

To assess the safety and efficacy of anakinra in pregnant FMF patients.

## Methods

Five FMF patients, treated with anakinra during pregnancy were monitored for side effects, fetal and maternal outcomes.

## Results

We present five FMF cases treated with Anakinra during pregnancy due to severe protracted febrile myalgia in 3, thrombocytopenia in 1 and amyloidosis in 1. One of these cases is among the 5 patients that have been previously reported (1). Throughout pregnancy no anakinra-related adverse event was observed in any of the patients. During the postpartum period one patient had an incision-site infection and the baby of the patient with thrombocytopenia also developed low platelet count which resolved with IVIG therapy. Otherwise all patients delivered normal babies. One of the patients is still pregnant and expecting twins. All of the patients, except one with colchicine intolerance continued with daily prophylactic colchicine treatment. Anakinra was terminated shortly after birth with success in all. Pregnancy-related features are listed in Table 1.

**Table 1:** pregnancy-related features

Case	Maternal Age	Anakinra Relation to pregnancy	USGs	Weeks at delivery or currrent gestational age	Gender of the baby/fetus	Mode of the delivery	1^st^minute APGAR	Follow-up duration after birth (months)	Complications after birth
1	33	started at 21st GW and used continiously until birth	normal	Birth at 36th GW	boy	C/S	8	32	No

2	28	started at 12th GW and used until birth	normal	Birth at 40th GW	girl	vaginal	10	20	No

3	31	started at 12th GW and used until birth	normal	Birth at 38th GW	boy	C/S	6	2	Methicillin-Sensitive Staphylococcus Aureus incision-site infection (treated with Tygecycline) in mother

4	24	started at 15th GW and used until birth	normal	Birth at 38th GW	boy	Vaginal	8	2	Low thrombocyte count in the baby at birth (23,000/mm3); resolved after 3 infusions of IVIG (269,000/mm3)

5	33	started at 16th GW, still using	normal	At 20th GW	Expecting 2 girls				

## Conclusions

Anakinra promises to be a safe alternative in pregnant FMF patients who are unresponsive or intolerant to colchicine. It can be administered transiently only during pregnancy and stopped after delivery.
